# High Seroprevalence of *Leptospira* Exposure in Meat Workers in Northern Mexico: A Case-Control Study

**DOI:** 10.14740/jocmr2463w

**Published:** 2016-01-26

**Authors:** Cosme Alvarado-Esquivel, Jesus Hernandez-Tinoco, Luis Francisco Sanchez-Anguiano, Agar Ramos-Nevarez, Sandra Margarita Cerrillo-Soto, Leandro Saenz-Soto, Lucio Martinez-Ramirez

**Affiliations:** a Biomedical Research Laboratory, Faculty of Medicine and Nutrition, Juarez University of Durango State, Durango, Mexico; b Institute for Scientific Research “Dr. Roberto Rivera Damm”, Juarez University of Durango State, Durango, Mexico; c Clinica de Medicina Familiar, Instituto de Seguridad y Servicios Sociales de los Trabajadores del Estado, Predio Canoas S/N, 34079 Durango, Mexico

**Keywords:** *Leptospira*, Case-control study, Epidemiology, Seroprevalence, Meat workers, Mexico

## Abstract

**Background:**

The seroepidemiology of *Leptospira* infection in workers occupationally exposed to raw meat has been poorly studied. This work aimed to determine the association between *Leptospira* exposure and the occupation of meat worker, and to determine the seroprevalence association with socio-demographic, work, clinical and behavioral characteristics of the meat workers studied.

**Methods:**

We performed a case-control study in 124 meat workers and 124 age- and gender-matched control subjects in Durango City, Mexico. Sera of cases and controls were analyzed for anti-*Leptospira* IgG antibodies using a commercially available enzyme immunoassay. Data of meat workers were obtained with the aid of a questionnaire. The association of *Leptospira* exposure with the characteristics of meat workers was analyzed by bivariate and multivariate analyses.

**Results:**

Anti-*Leptospira* IgG antibodies were found in 22 (17.7%) of 124 meat workers and in eight (6.5%) of 124 controls (OR = 3.12; 95% CI: 1.33 - 7.33; P = 0.006). Seroprevalence of *Leptospira* infection was similar between male butchers (17.6%) and female butchers (18.2%) (P = 1.00). Multivariate analysis of socio-demographic, work and behavioral variables showed that *Leptospira* exposure was associated with duration in the activity, rural residence, and consumption of snake meat and unwashed raw fruits.

**Conclusions:**

This is the first case-control study of the association of *Leptospira* exposure with the occupation of meat worker. Results indicate that meat workers represent a risk group for *Leptospira* exposure. Risk factors for *Leptospira* exposure found in this study may help in the design of optimal preventive measures against *Leptospira* infection.

## Introduction

Bacteria of the genus *Leptospira* cause a disease known as leptospirosis [1]. This disease is a worldwide zoonosis [2, 3]. *Leptospira* is excreted in the urine of infected animals [1]. Humans become infected with *Leptospira* by direct or indirect contact with infected animals and their urine or by contact with contaminated water and soil [1]. A number of animals can be infected with *Leptospira*, i.e., swine [2], sheep [4, 5], cows [1], rats, dogs [1, 6], and horses [7]. *Leptospira* infection does not only occur in domestic animals but also in wild and peri-domestic animals including deer [8] and other mammals, birds, and reptiles [9]. Infection with *Leptospira* is usually asymptomatic [10]. Clinical manifestations of leptospirosis include influenza-like symptoms, pulmonary hemorrhage [11], and renal and liver failure [12]. *Leptospira* infection in pregnant women may lead to fetal and maternal morbidity and mortality [13].

Very little is known about the seroepidemiology of *Leptospira* infection in workers occupationally exposed to raw meat. Studies in New Zealand have revealed seroprevalences of *Leptospira* infection of 10.2% in meat inspectors [14], and 6.2% in meat workers [15]. In a study in Italy, researchers found an 11.76% seroprevalence of *Leptospira* infection in meat workers [16], whereas in a study in Tanzania, abattoir workers had a 17.1% seroprevalence of *Leptospira* infection [17]. To the best of our knowledge, there is no case-control study about the association of *Leptospira* infection with the occupation of meat worker. In addition, we are not aware of any survey about *Leptospira* infection in meat workers in Mexico. Therefore, we sought to determine the association of *Leptospira* exposure with the occupation of meat worker in Durango City, Mexico and to determine the socio-demographic, clinical, work and behavioral characteristics of meat workers associated with *Leptospira* exposure.

## Materials and Methods

### Workers occupationally exposed to raw meat and controls

We performed an age- and gender-matched case-control study using serum samples from recent studies about the seroepidemiology of *Toxoplasma gondii* infection in Durango City, Mexico [18, 19]. Cases included 124 meat workers, and controls included 124 subjects without an occupation of meat worker. Sera from all participants were analyzed for the presence of anti-*Leptospira* IgG antibodies. Meat workers included in the study were those who have worked as butchers in abattoirs or butcher’s shops for at least 6 months, aged 16 years and older, and who accepted to participate in the study. None of the following characteristics of meat workers was a restrictive criterion for enrollment: gender, socio-economic status, or educational level. Fifty-nine meat workers were enrolled in 35 private butcher’s shops, 35 in a federal abattoir and 30 in a municipal abattoir. Meat workers (21 females and 103 males) were aged 16 - 71 years old (mean 38.5 ± 13.2 years). Controls were randomly selected from the general population of Durango City. They had occupations other than meat worker and were matched with cases by age and gender. We included one control for each case. The control group included 124 subjects (21 females and 103 males) aged 16 - 72 years (mean: 38.85 ± 13.68 years). The mean age in controls was comparable to that in meat workers (P = 0.69).

### Characteristics of meat workers

Socio-demographic, clinical, work and behavioral data of meat workers were obtained from previously submitted questionnaires [18]. Socio-demographic data included age, gender, birthplace, residence, educational level, and socio-economic status. Clinical data were current suffering from any disease, history of blood transfusion, and presence of visual impairment. Work data included duration (years) in the activity, frequency of contact with raw meat, habitual use of safety practices (use of hand gloves, face masks, and glasses), history of splashes at face with blood or raw meat, injuries with sharp material at work, and eating when working. Behavioral data were raising farm animals, foreign traveling, consumption of meat (pork, beef, goat, lamb, boar, chicken, turkey, pigeon, duck, rabbit, venison, squirrel, horse, opossum, snake or other), consumption of raw or undercooked meat, untreated water, unwashed raw vegetables and fruits, and soil contact.

### Detection of anti-*Leptospira* IgG antibody

Stored serum samples from cases and controls were analyzed for anti-*Leptospira* IgG antibodies by a commercially available enzyme immunoassay “*Leptospira* IgG ELISA test” kit (Diagnostic Automation Inc., Calabasas, CA). Seroreactivity to anti-*Leptospira* IgG antibodies was considered when an absorbance reading equal to or higher than 0.5 optical density (OD) units in a serum sample was found. Weak and strong seroreactivities of samples were considered when absorbance readings between 0.5 and 1.0 OD units and higher than 1.0 OD were found, respectively. Immunoassays were performed following the manufacturer’s instructions. According to the kit’s insert, the immunoassay used has a sensitivity of 100% and a specificity of 100%. Positive and negative controls were included in each run.

### Statistical analysis

Results were analyzed with the software Epi Info version 7, and SPSS version 15.0 (SPSS Inc. Chicago, IL). For calculation of the sample size, we used a 95% confidence level, a power of 80%, a 1:1 proportion of cases and controls, a reference seroprevalence of 5.6% [20] as the expected frequency of exposure in controls, and an odds ratio (OR) of 3.5. The result of the sample size calculation was 117 cases and 117 controls. To compare age values among cases and controls, the paired Student’s *t*-test was used. We used the Pearson’s Chi-square test or the two-tailed Fisher’s exact test (when cell values were less than 5) for comparison among groups. The association of *Leptospira* seropositivity with the socio-demographic, clinical, work and behavioral characteristics of meat workers was assessed by bivariate and multivariate analysis. Only variables with a P value ≤ 0.05 obtained in the bivariate analysis were included in the multivariate analysis. The Hosmer-Lemeshow goodness of fit test was used to assess the fitness of our regression model. OR and 95% confidence interval (CI) were calculated by logistic regression with the Enter method. A P value < 0.05 was considered statistically significant.

### Ethical aspects

This study was approved by the Ethics Committee of the Instituto de Seguridad y Servicios Sociales de los Trabajadores del Estado in Durango City, Mexico.

## Results

Anti-*Leptospira* IgG antibodies were found in 22 (17.7%) of 124 meat workers and in eight (6.5%) of 124 controls (OR = 3.12; 95% CI: 1.33 - 7.33; P = 0.006). High (OD >1.0) anti-*Leptospira* IgG antibody levels were found in six (4.8%) of 124 meat workers and in two (1.6%) of 124 controls (OR = 3.10; 95% CI: 0.61 - 15.67; P = 0.28). Seroprevalence results were stratified by gender. Anti-*Leptospira* IgG antibodies were found in four (18.2%) of 22 female meat workers and in one (4.5%) of 22 female controls (OR = 4.66; 95% CI: 0.47 - 45.62; P = 0.34). Whereas anti-*Leptospira* IgG antibodies were found in 18 (17.6%) of 102 male meat workers and in seven (6.9%) of 102 male controls (OR = 2.90; 95% CI: 1.15 - 7.30; P = 0.01). Seroprevalence of *Leptospira* infection was similar between male butchers (17.6%) and female butchers (18.2%) (P = 1.00).

Concerning the socio-demographic, clinical, work and behavioral characteristics in the meat workers studied, the variables duration in the activity, residence area, and consumption of snake meat and unwashed raw fruits had P values < 0.05 by bivariate analysis. Other socio-demographic and behavioral characteristics including age, gender, birthplace, educational level, socio-economic status, raising farm animals, traveling, consumption of meat other than snake meat, consumption of raw or undercooked meat, untreated water, unwashed raw vegetables, and soil contact had P values ≥ 0.05 by bivariate analysis. None of the clinical characteristics studied including presence of any disease, history of blood transfusion, and presence of visual impairment had P values < 0.05 by bivariate analysis. In addition, other work characteristics, i.e., frequency of contact with raw meat, habitual use of safety practices, history of splashes at face with blood or raw meat, injuries with sharp material at work, and eating when working had P values < 0.05 by bivariate analysis. A correlation of the seroprevalence of *Leptospira* infection with a selection of characteristics of meat workers is shown in Table 1. Multivariate analysis of socio-demographic, work and behavioral variables with P values < 0.05 obtained by bivariate analysis showed that *Leptospira* exposure was positively associated with duration in the activity, rural residence, and consumption of snake meat and unwashed raw fruits. Table 2 shows the results of the multivariate analysis. The result of the Hosmer-Lemeshow test (P = 0.17) suggested a good fitting of our regression model.

**Table 1:** Bivariate Analysis of a Selection of Characteristics of Meat Workers and Seroprevalence of *Leptospira* Exposure

Characteristic	No. of subjects tested*	Positive ELISA results, No. (%)	P value
Age groups (years)			
30 or less	39	4 (10.3)	0.39
31 - 50	59	12 (20.3)	
> 50	25	5 (20)	
Residence area			
Urban or suburban	91	9 (9.9)	0.0008
Rural	32	12 (37.5)	
Educational level			
No education	4	2 (50.0)	0.13
Education	118	19 (16.1)	
Socio-economic status			
Low	42	6 (14.3)	0.42
Medium	74	15 (20.3)	
National trips			
Yes	72	9 (12.5)	0.08
No	49	12 (24.5)	
Goat meat consumption			
Yes	43	10 (23.3)	0.16
No	75	10 (13.3)	
Sheep meat consumption			
Yes	90	18 (20)	0.17
No	32	3 (9.4)	
Snake meat consumption			
Yes	12	6 (50)	0.007
No	112	16 (14.3)	
Consumption of raw or undercooked meat			
Yes	13	1 (7.7)	0.46
No	111	21 (18.9)	
Consumption of unwashed raw vegetables			
Yes	45	10 (22.2)	0.14
No	74	9 (12.2)	
Consumption of unwashed raw fruits			
Yes	77	17 (22.1)	0.03
No	42	3 (7.1)	
Untreated water			
Yes	82	16 (19.5)	0.21
No	38	4 (10.5)	
Soil contact			
Yes	85	17 (20)	0.21
No	37	4 (10.8)	
Duration in the activity			
Up to 10 years	64	7 (10.9)	0.04
More than 10 years	51	13 (25.5)	
Safety practices (gloves, mask, glasses)			
Yes	11	4 (36.4)	0.11
No	105	17 (16.2)	
Splash of blood or raw meat at face			
Yes	88	18 (20.5)	0.15
No	32	3 (9.4)	
Injuries at work			
Yes	111	19 (17.1)	0.65
No	9	2 (22.2)	
Visual impairment			
Yes	80	16 (20)	0.16
No	40	4 (10)	

*Sums may not add up to 124 because of some missing values.

**Table 2:** Results of the Multivariate Regression Analysis

Variables	P value	Odds ratio	95% confidence interval
Rural residence	0.01	4.29	1.29 - 14.20
Duration in the activity	0.03	3.87	1.13 - 13.22
Snake meat consumption	0.01	7.20	1.48 - 34.91
Consumption of unwashed raw fruits	0.03	6.68	1.16 - 38.55

## Discussion

Surveys of *Leptospira* infection in workers with occupational exposure to raw meat have been performed in a limited number of countries. In addition, the magnitude of the association of *Leptospira* seropositivity with the occupation of meat worker has not been previously evaluated by an age- and gender-matched case-control study. Therefore, this study aimed to determine the association of *Leptospira* seropositivity and the occupation of meat worker in the northern Mexican city of Durango. We found that seroprevalence of *Leptospira* exposure was significantly higher in meat workers than in control subjects. Male and female meat workers had a similar seroprevalence of *Leptospira* exposure. The seroprevalence found in meat workers (17.7%) is also higher than the 4.4% seroprevalence found in waste pickers in the same Durango City [20]. On the other hand, the seroprevalence found in meat workers is comparable with the 15.6% seroprevalence reported in the general population in rural Durango [21]. In an international context, the seroprevalence found in meat workers in Durango is higher than the 10.2% seroprevalence reported in meat inspectors in New Zealand [14], the 11.76% seroprevalence found in meat workers in Italy [16], and the 7% seroprevalence reported in slaughterhouse workers in Colombia [22]. The seroprevalence found in this study is comparable with the 17.1% found in abattoir workers in Tanzania [17]. In contrast, the seroprevalence found in meat workers in Durango is lower than the 29.2% seroprevalence of *Leptospira* infection found in butchers/abattoir workers in Nigeria [23].

We searched for factors associated with *Leptospira* infection in meat workers. Interestingly, multivariate analysis showed that *Leptospira* exposure was associated with duration in the activity of meat worker. This finding is consistent with that found in a recent study in New Zealand where researchers found that the time worked in the meat industry was a risk factor for *Leptospira* infection [24]. Multivariate analysis also showed that *Leptospira* exposure was associated with the following factors: rural residence, and consumption of snake meat and unwashed raw fruits. We have recently studied the seroepidemiology of *Leptospira* infection in the general population in rural Durango and found a high (15.6%) seroprevalence of *Leptospira* infection [19]. However, meat workers with rural residence had a more than two-fold higher seroprevalence (37.5%) of *Leptospira* infection than the one reported in the general population in rural Durango. This fact suggests that other factors, i.e., contact with raw meat might have contributed to the increasing seroprevalence. It is likely that people living in rural communities have more contact with animals than people do in urban communities. In fact, rural work (raising animals and agriculture) was a risk factor for *Leptospira* seropositivity in a survey in Colombia [22]. Poor education of the head of the family was a risk factor for *Leptospira* infection in rural Durango [21]. Intriguingly, *Leptospira* seropositivity was associated with consumption of snake meat. To the best of our knowledge, there is no previous report of this association. The epidemiological link of *Leptospira* infection and consumption of snake meat is supported by the fact that infection with *Leptospira* has been demonstrated in snakes. Seroreactivity to several *Leptospira* species was found in six of 22 snakes sampled in Slovenia [25]. In addition, seropositivity to *Leptospira* was found in 13 of 18 snakes in Argentina [26]. In contrast, none of 10 free-ranging Venezuelan anacondas had anti-*Leptospira* antibodies [27]. In a study in Brazil, researchers found not only antibodies to *Leptospira* in snakes but also found DNA of *Leptospira* by polymerase chain reaction in kidney and liver of snakes [28]. Further studies to confirm the association of *Leptospira* infection and consumption of snake meat are needed. In our study, *Leptospira* exposure was associated with consumption of unwashed raw fruit. This finding reflects poor hygiene practices. Fruits may be contaminated with *Leptospira* when contact soil contaminated with urine of *Leptospira*-infected rodents and other animals. In fact, fruits have been related with leptospirosis. A case of leptospirosis with acute renal failure in a man who ate unwashed raw fruits was reported [29]. A case of Weil’s syndrome with bone marrow involvement in a 65-year-old man who in recent weeks had collected walnuts in a region with infestation of rats was reported in Germany [30]. Further studies on the association of *Leptospira* infection and consumption of unwashed raw fruits are needed.

### Conclusions

This is the first case-control study of the association of *Leptospira* exposure with the occupation of meat worker, and of the association of *Leptospira* exposure with the consumption of snake meat. Results indicate that meat workers represent a risk group for *Leptospira* exposure. Risk factors for *Leptospira* exposure found in this study may help in the design of optimal preventive measures against *Leptospira* infection.
